# Supplementary figures and images for: Green Power Grids: How Energy from Renewable Sources Affects Networks and Markets
Source: PLoS One. 2015 Sep 3;10(9):e0135312. doi: 10.1371/journal.pone.0135312 (PMC4559395; doi:10.1371/journal.pone.0135312)

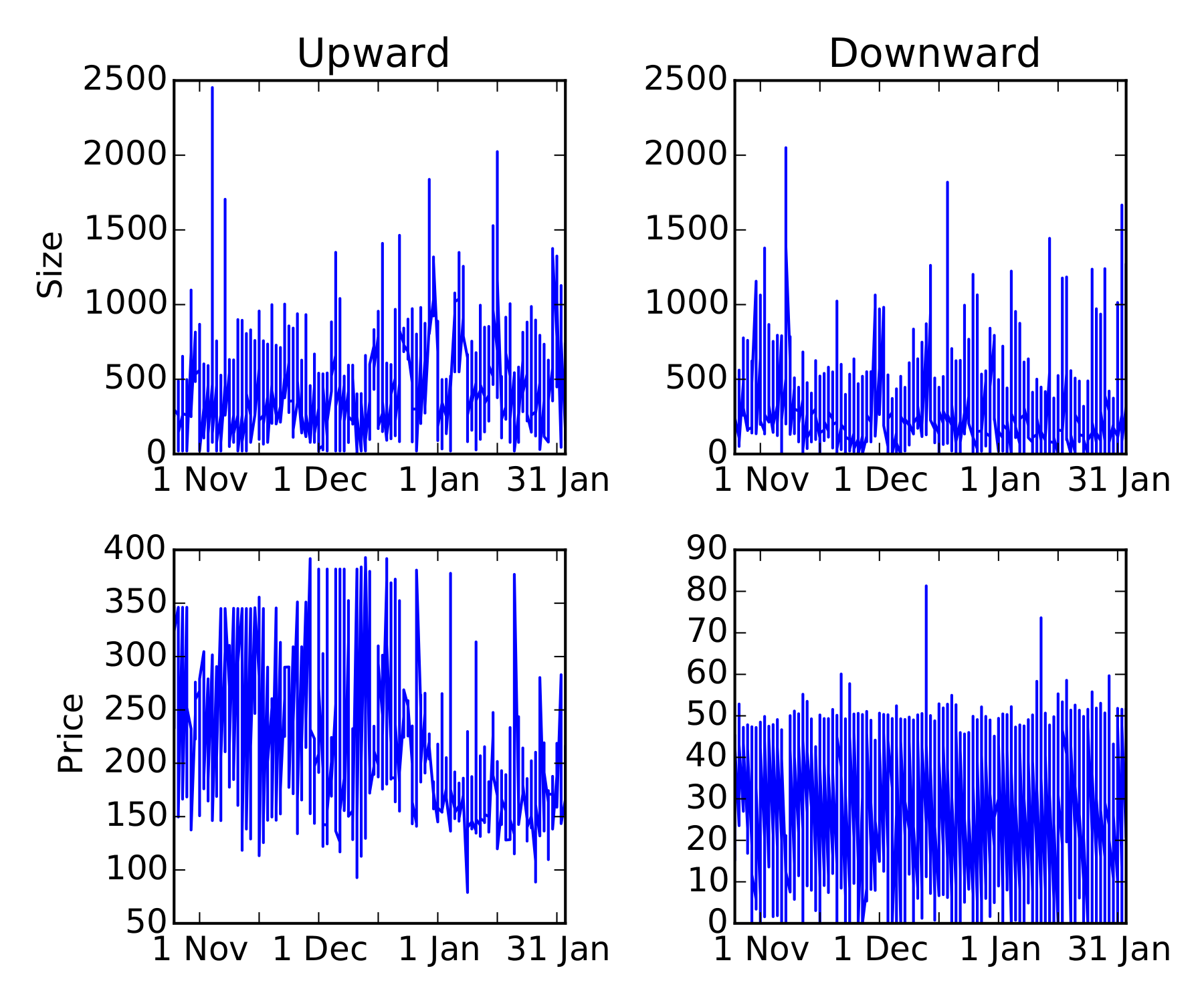

Supplement: S1 Fig — (TIF) [file pone.0135312.s001.tif]

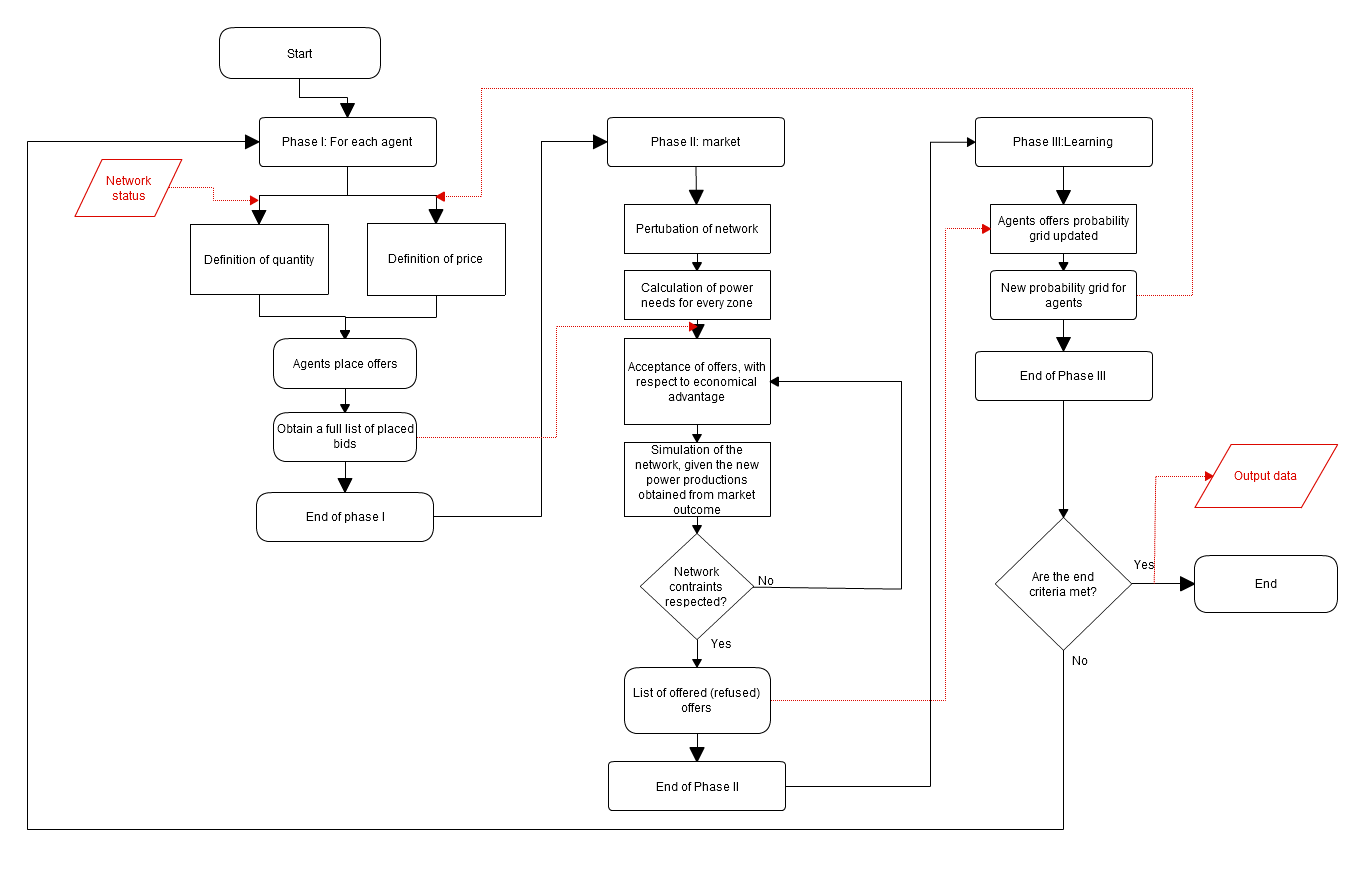

Supplement: S2 Fig — Notice that the couplings among the electric network and the electricity market must be taken into account during the simulation. (TIF) [file pone.0135312.s002.tif]
